# Membrane Ruffles: Composition, Function, Formation and Visualization

**DOI:** 10.3390/ijms252010971

**Published:** 2024-10-12

**Authors:** Guiqin Yan, Jie Zhou, Jiaxin Yin, Duolan Gao, Xiaohai Zhong, Xiaoyan Deng, Hongyan Kang, Anqiang Sun

**Affiliations:** Key Laboratory of Biomechanics and Mechanobiology (Beihang University), Ministry of Education, Beijing Advanced Innovation Center for Biomedical Engineering, School of Biological Science and Medical Engineering, Beihang University, Beijing 100083, China; yanguiqin@buaa.edu.cn (G.Y.); 21101037@buaa.edu.cn (J.Z.); yinjiaxin0906@163.com (J.Y.); gaoduolan@126.com (D.G.); 18374137@buaa.edu.cn (X.Z.); dengxy1953@buaa.edu.cn (X.D.)

**Keywords:** membrane ruffles, cell motility, macropinocytosis, viscosity sensing, growth factor, calcium, optic imaging

## Abstract

Membrane ruffles are cell actin-based membrane protrusions that have distinct structural characteristics. Linear ruffles with columnar spike-like and veil-like structures assemble at the leading edge of cell membranes. Circular dorsal ruffles (CDRs) have no supporting columnar structures but their veil-like structures, connecting from end to end, present an enclosed ring-shaped circular outline. Membrane ruffles are involved in multiple cell functions such as cell motility, macropinocytosis, receptor internalization, fluid viscosity sensing in a two-dimensional culture environment, and protecting cells from death in response to physiologically compressive loads. Herein, we review the state-of-the-art knowledge on membrane ruffle structure and function, the growth factor-induced membrane ruffling process, and the growth factor-independent ruffling mode triggered by calcium and other stimulating factors, together with the respective underlying mechanisms. We also summarize the inhibitors used in ruffle formation studies and their specificity. In the last part, an overview is given of the various techniques in which the membrane ruffles have been visualized up to now.

## 1. Introduction of Membrane Ruffles

Membrane ruffling, namely cell ruffling, is a complex process that is regulated by multiple molecules such as p85, phospholipase A2, and cholesterol [1]. In the process of membrane ruffling, characteristic structures called membrane ruffles, which are fold-like protrusions mainly composed of actin filaments covered with certain kinds of proteins on the surface of various kinds of cells like kidney cells, macrophages, and muscle cells, are generated and disassembled rapidly. It has been demonstrated that membrane ruffles may have several important biological functions. For example, they can become sensors of extracellular fluid viscosity, therefore controlling cell locomotion and force generation in the viscous microenvironment, and they are the foundation of lamellipodia formation in cell migration [2]. Moreover, they are also essential for C3bi-particle binding during Mac-1-mediated phagocytosis in macrophages [3]. The membrane ruffles can be divided into circular ruffles (type I membrane ruffles) or linear ruffles (type II membrane ruffles) in accordance with their morphological differences; dorsal or peripheral membrane ruffles with respect to their location on the cell surface; and epidermal growth factor (EGF)-stimulated, platelet-derived growth factor (PDGF)-induced, phorbol ester (PMA)-induced, and macrophage colony-stimulating factor (M-CSF)-induced membrane ruffles if considering the differences in stimulating factors.

In the present review, the current knowledge on the characteristic structure and molecular composition of both linear and circular membrane ruffles, and their multiple biological functions, were discussed. In the later section, growth factor-dependent and -independent initiation of the formation of membrane ruffles and the underlying mechanistic insights were investigated. Additionally, we outlined the inhibitors employed in ruffle formation studies and discuss their specificity. Finally, we reviewed the visualization and quantification technologies for membrane ruffles that have been reported up until now.

## 2. Structure and Molecular Composition of Membrane Ruffles

### 2.1. Structure of Linear Membrane Ruffles

**Figure 1:**
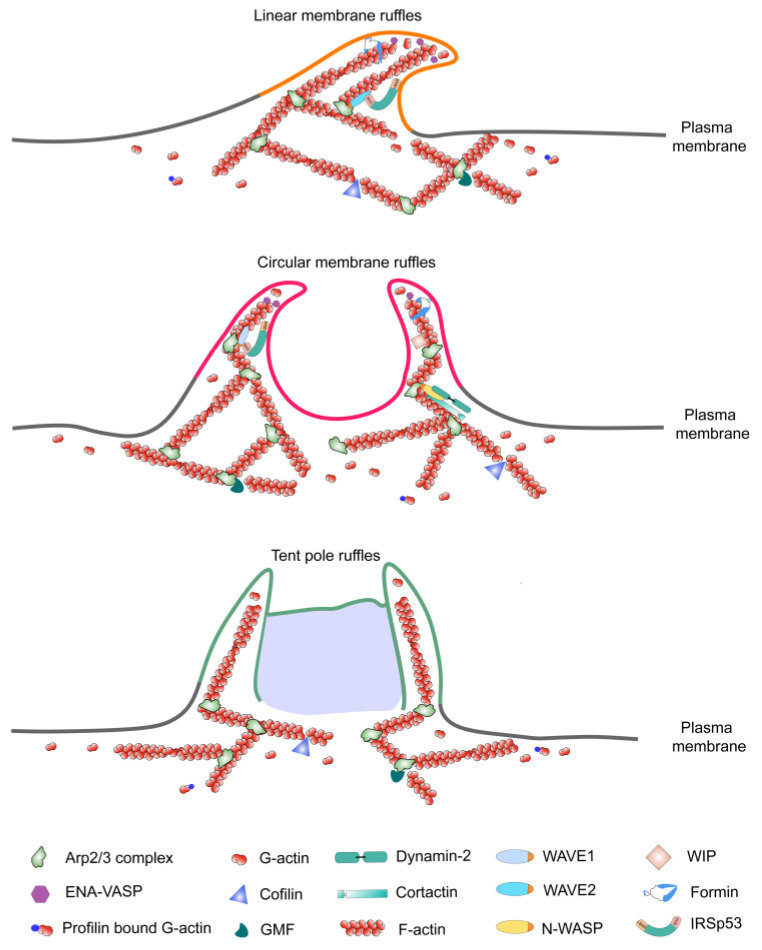
Structure and composition of membrane ruffles. The linear ruffles assemble at the leading edge of a cell membrane, with columnar spike-like and veil-like structures. Circular dorsal ruffles have no supporting columnar structures but have veil-like structures, connecting from end to end, that present an enclosed ring-shaped circular outline. Tent pole ruffles are supported by two distinct filamentous extensions that stand erect on the cell surface at the outer edge of the fold, acting like “tent poles” and holding up the large F-actin sheet which serves as the veil of the fold between them. Some clarified actin-binding proteins associated with membrane ruffles are illustrated in the figure.

There are significant structural differences between linear membrane ruffles and circular membrane ruffles. The composition of linear membrane ruffles features a large number of supporting columnar spike-like structures and veil-like structures, which are arranged linearly, making the membrane at the leading edge fold or crease backward and sometimes wave toward the cell center (Figure 1). The formation of linear membrane ruffles usually occurs in proximity to or at the cell periphery, and the process finishes within 1 min [4]. Afterward, they are persistently in the cycle of formation and disassembly. The fact that reagents that facilitate or inhibit the depolymerization of microtubules, such as nocodazole and microtubule-stabilizing reagents like Taxol, can both inhibit the generation of linear membrane ruffles suggests that microtubules may play important roles in the formation of linear membrane ruffles [5]. Although the exact role of microtubules remains unclear, considering there is no need for microtubules to support the whole structure, microtubules may act as signal molecules involved in protein kinase pathways in the process of membrane ruffle assembly.

### 2.2. Structure of Circular Membrane Ruffles

In contrast, circular membrane ruffles have no supporting columnar structures. The veil-like structures connecting from end to end are erect vertically on the dorsal surface of the plasma membrane, presenting an enclosed ring-shaped circular outline (Figure 1). Unlike the persistent formation and disassembly cycle of linear membrane ruffles, circular membrane ruffles only form once upon stimulation and then disappear within 5 to 30 min [6]. Nocodazole and Taxol have no effect on the formation of circular ruffles, which means that they show no dependence on microtubules [5].

### 2.3. Tent Pole Ruffles—A Unique Structure

With novel technologies such as lattice light-sheet microscopy (LLSM), researchers have discovered a unique structure in the observation of dorsal membrane ruffles and found this unique tent pole structure was involved in macropinosome formation. Two distinct filamentous extensions stand erect on the cell surface at the outer edge of the fold, acting like “tent poles”, holding up the large F-actin sheet which serves as the veil of the fold between them (Figure 1). Thus, the two protrusions are named tent poles. The tent pole is a highly dynamic structure that cannot be observed by traditional techniques because the assembling and disassembling of it is extremely rapid [7]. During the process of macropinosome formation induced by lipopolysaccharide (LPS) in RAW264.7 macrophage cells, filopodial-like tent poles extended at some “hotspots” on the cell surface, which then initiated the formation of a membrane/F-actin veil between the extended tent poles. Thereafter, the erected tent poles converged and the ruffles began to circulate. The crossing over and twisting of the tent poles finally led to the collapse of the membrane veil and the formation of macropinosome. Although the mechanism of the formation and regulation of tent poles has not been fully discovered, this process can be regulated by Rab13 (a ruffle-associated small GTPase), as shown by researchers who identified that after silencing Rab13, the tent pole ruffle formation and dynamics were affected, coinciding with the impairment of macropinosome production [8].

### 2.4. Composition of Membrane Ruffles

Almost all membrane ruffles contain densely packed arrays of actin filaments which assemble a net with thin threads. These arrays are associated with a distinct set of actin-binding proteins, such as Arp2/3-mediated actin nucleation, branching, and elongation proteins and actin filament cross-linkers [9,10]. The coordination of these proteins regulates the formation and growth of membrane ruffles (Figure 1).

#### 2.4.1. Actin-Nucleating Proteins

The mechanism for initiating assembly of actin filaments involves de novo nucleation, where proteins like cordon-bleu (Cobl) and junction-mediating regulatory protein (JMY) use their multiple G-actin-binding WH2 domains to form actin trimers and nucleate actin filaments. Cobl, a highly expressed protein in the brain, has been shown to promote actin assembly and induce ruffling in COS-7 cells [11]. Additionally, Cobl-like proteins bind G-actin via their WH2 domains and interact with the Src homology (SH3) domain of actin-binding protein-1 (Abp1), collaborating to assemble filaments and form membrane ruffles, which is crucial in neuronal development and morphogenesis [12,13]. JMY facilitates the nucleation of linear actin filaments via its WH2 domains and activates Arp2/3-mediated branching via its C-terminal WCA domain [14,15,16]. In metastatic cells, JMY has been linked to reduced E-cadherin stability, promoting migration [15]. However, their role in ruffle formation is less understood.

Cofilin, an actin filament-severing protein, facilitates the turnover of actin monomers which are essential for the further assembly of primer actin filaments [17,18]. The activation of cofilin (dephosphorylation at Ser-3) severs pre-existing filaments and exposes barbed ends for Arp2/3-mediated network reconstruction, further stimulating ruffle formation and macropinocytosis [19]. Additionally, GMF, a member of the cofilin family, can specifically sever the junction between Arp2 and the actin filament, leading to the debranching of aged ADP-containing actin filaments [20,21]. It has been reported that GMF associates with membrane ruffles and promotes migration by enhancing the recycling of branched actin filaments [22,23].

#### 2.4.2. Actin Branching Proteins

The Wiskott-Aldrich syndrome protein (WASP) family mediates actin filament branching. WASP and neural-WASP (N-WASP) proteins both contain the carboxy-terminal verpolin homology, cofilin homology, and acidic (VCA) domain, where binding of G-actin occurs to initiate actin network assembly [24]. The WASP-family verprolin-homologous proteins (WAVE) are key activators of the Arp2/3 complex, with the subfamily comprising WAVE1, WAVE2, and WAVE3. All WAVE proteins contain VCA regions at their C-terminal, which are essential for inducing membrane ruffle formation [6]. WAVE1 is notably involved in the regulation of dorsal ruffles, while WAVE2 is primarily associated with peripheral ruffles [25,26,27]. WASP-interacting protein (WIP) can independently bind to F-actin and is crucial for regulating the formation of various actin structures. High expression levels of WIP promote the formation of dorsal ruffles [28]. c-Abl interacts with WAVE2, phosphorylating it to activate actin remodeling, which is important for membrane ruffle formation and cell spreading [29]. Furthermore, the Scar/WAVE complex could also activate the Arp2/3 complex, playing a crucial role in membrane ruffle formation during macropinocytosis [30].

Cortactin has been reported to be highly enriched in lamellipodia and membrane ruffles, where it plays a key role in stabilizing actin branches formed by the Arp2/3 complex [31]. Cortactin also participated in signal transduction. For instance, upon activation of tyrosine kinase receptors, cortactin is phosphorylated on its tyrosine residues, thus playing an important role in microfilament–membrane interactions as well as transducing signals from the cell surface to the cytoskeleton [32]. Depletion of cortactin reduces lamellipodial persistence and impairs cell migration [33]. The N-WASP–Arp2/3–cortactin–dynamin complex was found to be colocalized with the CDR ring, which is regulated by small GTPases [6,34].

#### 2.4.3. Actin Elongation Proteins

The Ena/VASP protein family, consisting of Mena, VASP, and EVL, shares a conserved tripartite domain structure: a proline-rich central domain (profilin-binding site), an EVH1 domain, and an EVH2 domain (F-actin- and G-actin-binding sites) [35]. Ena/VASP proteins localize to the tips of filopodia, lamellipodia edges, peripheral and circular dorsal ruffles, and focal adhesions [36]. By binding the barbed ends of filaments, Ena/VASP promotes filament elongation, generating high F-actin levels [10,22]. It has been suggested that an imbalance between actin branching and elongation leads to ruffling in the cell leading edge [22,37]. Specifically, when actin elongation dominates over actin branching, for example, overexpression of Ena/VASP produces longer, less branched lamellipodia that are prone to lifting backwards during ruffling, reducing migration efficiency. Conversely, depletion of Ena/VASP proteins results in shorter, more highly branched actin filaments, leading to slower but more persistent lamellipodial protrusions [38].

Formins, including the Dia (mDia1, mDia2, mDia3) and FMNL (FMNL1, FMNL2, FMNL3) subfamilies, contain FH1 and FH2 domains. The FH1 domain binds profilin–actin, while the FH2 domain promotes filament elongation through a processive association with barbed ends. FMNL2/3 are essential for nucleating and elongating actin filaments, promoting smooth protrusions and efficient migration [39]. In addition, mDia2 is crucial for filopodia formation and also contributes to lamellipodial persistence.

Profilin catalyzes the exchange of ADP for ATP on G-actin, aiding in the delivery of profilin-bound G-actin to growing actin filament ends, particularly at barbed ends, promoting elongation. Profilin is reported to stabilize actin filaments and inhibit membrane ruffling in non-neuronal cells by enhancing submembranous actin network density [40]. However, recent studies have also shown that profilin 1, a profilin isoform, interacts with ENA/VASP and formins to more efficiently assemble and elongate filaments that contribute to cell protrusion [41].

CARMIL promotes Arp2/3-mediated actin polymerization by inhibiting capping proteins from binding to actin filament barbed ends, allowing continuous filament growth [42]. It exists in three isoforms, CARMIL1, CARMIL2, and CARMIL3, of which CARMIL1 has been studied in more detail and is known to be localized to lamellipodia and macropinosomes [43]. Knockdown of CARMIL1 results in reduced lamellipodial actin, slower protrusion speed, and significant inhibition of cell migration and macropinocytosis. CARMIL may serve as a potential regulator of membrane ruffling, which needs to be further elucidated.

#### 2.4.4. Actin Cross-Linking Proteins

The actin filaments cross-linkers such as filamin and ezrin contribute to the organization and stabilization of membrane ruffles [44]. Filamin is a protein found in tissues such as smooth muscle, which can induce calcium-insensitive actin gelling, promote vertical branching of actin filaments, and bind actin filaments to glycoproteins on the membrane [45]. Ezrin is a membrane cytoskeletal connexon protein that is up-regulated in a variety of human tumors and can regulate cell–cell and cell–extracellular matrix adhesion through interaction with cell adhesion molecules [46]. During the process of cell locomotion in response to EGF stimulation, ezrin, phosphorylated by protein kinase C (PKC) in small ruffles at the edge of cells, was found together with myosin V and myosin VI in A431 cells [47].

Except for the actin and actin regulation proteins mentioned above, some other molecules could also be found colocalized to the membrane ruffles when cells execute specific biological functions. For instance, in the process of blood glucose regulation, glucose transporter type 4 (GLUT-4) can be inserted in the membrane ruffles of muscle cells with the presence of insulin [48]. In addition, after fibroblast growth factor (FGF) stimulation, the heat shock protein 27 (HSP27), localizing in lamellipodia and membrane ruffles, can be quickly phosphorylated on its serine residues and stimulate the accumulation of actin filaments, while the non-phosphorylation mutant inhibits cells’ response to FGF, suggesting that HSP27 may be a key component of the FGF signal transduction pathway regulating the microfilament dynamics [49]. IRSp53, a substrate protein of insulin receptor tyrosine kinase, has been identified as a critical mediator linking membrane and actin dynamics during membrane ruffle formation [50,51]. Its inverted bin-amphiphysin-rvs (I-BAR) domain binds to phosphatidylinositol 4,5-bisphosphate (PI(4,5)P2)-rich lipid regions, facilitating membrane bending, while the SH3 domain interacts with various actin regulatory proteins, such as WAVE, N-WASP, dynamin, and mDia1 [52], to promote actin polymerization. Furthermore, IRSp53 connects with Rac through its I-BAR domain and recruits WAVE2 via the SH3 domain [53]. The Rac1-IRSp53-WAVE2 signaling axis has been shown to regulate Ras-induced macropinocytosis and the formation of membrane ruffles [54].

## 3. The Biological Functions of Membrane Ruffles

### 3.1. In Cell Motility

**Figure 2:**
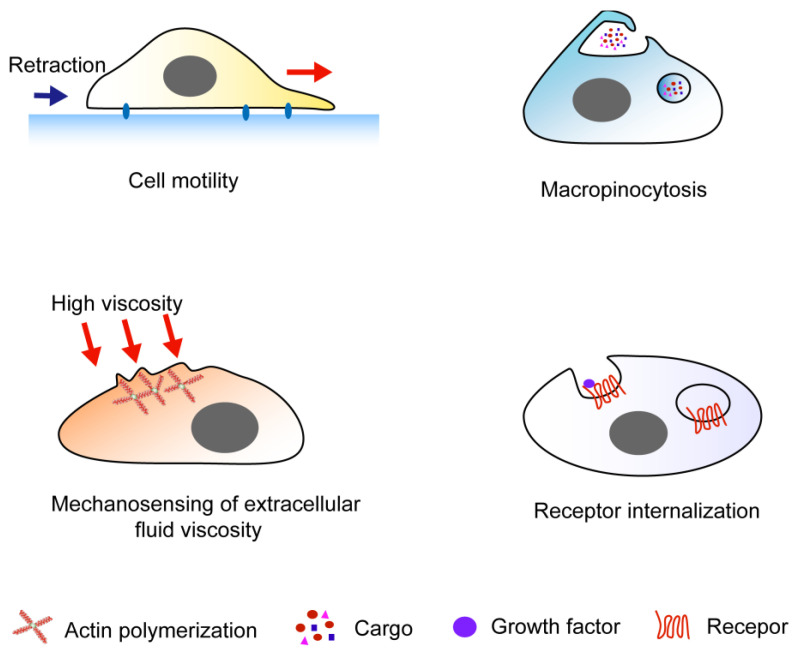
Biological functions of membrane ruffles. Linear membrane ruffles are involved in all these processes. Circular dorsal ruffles (CDR) are primarily involved in cell migration, macropinocytosis, and receptor internalization. Tent pole ruffles mainly function in macropinocytosis and receptor internalization.

In the initiation of cell migration, membrane ruffles are often found at the advancing front of a lamellipodium, serving as sites of actin polymerization (Figure 2). Therefore, membrane ruffles were viewed as a characteristic sign of actin reorganization in cell responses to chemotactic factors [55,56].

It has been reported by Borm et al. [44] that there was an inverse relationship between the ruffle frequency and cell migration, which means a dramatic increase in ruffling frequency will result in inefficient cell migration. Moreover, they concluded the formation of membrane ruffles is actually a consequence of inefficient integrin–ligand interaction which results in inefficient lamellipodia adhesion. This inverse relationship between membrane ruffle formation and cell migration has also been observed in a recent study by Pittman et al. [2].

In cell locomotion, Price et al. [57] observed membrane ruffles of monkey kidney cells (LLCMK 2) with a scanning electron microscope (SEM) and noticed that membrane ruffles seem to appear in all directions and then vanish in the plasma membrane rapidly, such that the total effect of membrane ruffling induces the orientation movement of cells. Interestingly, the contact of membrane ruffles with other cells or any solid or semi-solid object will stop the formation of ruffles, which is important to prevent the large deformation of the plasma membrane and maintain the cell shape. Most recently, Sitarska et al. [58] revealed how cells balance persistent self-propelled motion with adaptive behaviors to circumvent obstacles. They identified a curvature-sensitive BAR domain protein, sorting nexin 33 (Snx33), which inhibits actin polymerization and ruffling, playing pivotal roles in reading cell surface topography and plasma membrane biophysics to interpret their environment and make decisions (moving ahead or turning away).

### 3.2. In Macropinocytosis

Micropinocytosis is a kind of endocytic process in which a large amount of extracellular fluid and its content is internalized by forming dynamic and actin-driven plasma membrane protrusions, namely membrane ruffles [59] (Figure 2). This process is induced by growth factors such as M-CSF, EGF, and other stimuli in multiple cell types. In addition, in innate immune cells, a calcium-dependent constitutive micropinocytosis exists [60]. During the process of macropinocytosis, a large, heterogeneous vacuole structure called the macropinosome is generated as many membrane ruffles fuse at their distal margins, forming cups, and separating into the cytoplasm. Membranes ruffling-mediated macropinocytosis has been reported to be involved in a number of pathological conditions including Alzheimer’s disease, atherosclerosis, cancer, Parkinson’s disease, and nephrolithiasis [61,62,63,64,65].

Moreover, membrane ruffles are reported to be involved in the binding of C3bi-opsonized particles in activated macrophages. Upon stimulation, the expression of macrophage-1 antigen (Mac-1) is up-regulated and more Mac-1 can be located on the cell surface. The formation of ruffling promotes the accumulation of Mac-1 receptors and the recruitment of talin, thus promoting the aggregation and activation of integrins within membrane ruffles, which in turn promotes the adhesion of adjacent complement conditioning particles. This process seems to need the integrity of microtubules in cells since microtubule disruption inhibits membrane ruffle formation and surface Mac-1 expression. When the particle is attached to the surface of the macrophage, it folds under the bound particle to form a giant pyrosome, after which the particle slowly “sinks” into the cytoplasm for maturation. Recycling endosomes (macropinosomes), microtubules, and kinesin are necessary for this continuous internalization process [3]. It should be noted that during macropinosome formation, circular membrane ruffles can establish barriers to inhibit the diffusion of signaling intermediates, which could explain why signals essential for macropinosome formation are restricted to the domain of circular ruffles [66].

### 3.3. Receptor Internalization

Internalization of receptor tyrosine kinase (RTK) is essential in regulating growth factor-stimulated events, such as EGF-, PDGF-, and hepatocyte growth factor (HGF)-stimulated cell signaling events, and affects cell growth, mitosis, motility, and invasion (Figure 2). The most widely recognized RTK internalization machinery is mediated by clathrin-coated pits, the assembly of which also needs adaptor, scaffolding, dynamin, and some cytoskeleton proteins. However, in the 1970s, Haigler et al. [67,68] used ferritin-conjugated EGF in A431 cells and observed that some EGF particles located to regions of the cell membrane without occurrence of clathrin-mediated endocytosis, suggesting epidermal growth factor receptors (EGFRs) may be internalized in a clathrin-independent manner. Two decades later, Sigismund et al. [69] demonstrated the internalization of EGFRs was EGF-concentration-dependent. Briefly, at low concentrations (1.5 ng/mL), endocytosis of EGFRs was mainly clathrin-mediated, whereas at high concentrations (20–100 ng/mL), a clathrin-independent mode simultaneously emerged. Orth et al. [70] identified the next year that circular dorsal membrane ruffles can mediate another EGFR internalization mode. In addition to EGF, circular dorsal membrane ruffle-mediated receptor internalization was also observed in other growth factors’ stimulation, such as PDGF and HGF [6]. Moreover, the large GTPase dynamin and its binding partner cortactin, as well as some other proteins like Rac, Ras, Rab5, and the lipid and serine-threonine kinases phosphatidylinositol 3-kinase and p21-activated kinase-1, were all involved in the formation of CDRs [71]. Interestingly, fewer CDRs seem to form in pancreatic and prostate tumor cells such as BxPC3, PC3, HPAF, and PANC-1 compared with mouse fibroblasts and primary human fibroblasts, which may have significant effects on the receptor signaling and tumor cell growth [72].

### 3.4. Mechanosensing of Extracellular Fluid Viscosity

A recent study performed by Pittman et al. [2] suggested that multiple adherent cells, such as MDA-MB-231 breast cancer cells, use membrane ruffles to sense changes in extracellular fluid viscosity to trigger adaptive responses (Figure 2). Briefly, in a highly viscous medium, the formation of membrane ruffles was suppressed, which in turn enhanced integrin–substrate engagement, increased nascent adhesion growth, facilitated cell spreading, generated greater traction force, and enhanced focal adhesion turnover; ultimately, cells migrated almost two times faster. However, the hypothesis proposed by Pittman et al. was only limited to cells plated on a 2D surface. Whether cells embedded in a 3D microenvironment sense the fluid viscosity by using membrane ruffles is still unknown. Moreover, in the physiological condition, the microenvironment of tissue cells is likely to be more viscoelastic. Could this hypothesis be extended to viscoelasticity in cells? All these questions remain to be answered in the future study.

### 3.5. Unfolding of Membrane Ruffles in Response to Physiologically Compressive Loads to Protect Cells from Death

Moo et al. [73] demonstrated that chondrocytes in explanted bovine cartilage will expand their membrane surface through unfolding of the membrane ruffles following exposure to compressive strains ranging from 0% to 50%, which is essential for the cells to avoid membrane rupture and protect them from death. Although the underlying mechanism is still unknown, the load-induced membrane unfolding may play important roles in mechanotransduction through stretch-activated ion channels in the cell membrane or the deformation of the cytoskeleton to regulate cell metabolism [74]. It should be noted that unfolding of membrane ruffles is not the sole mechanism that chondrocytes use to protect membranes from excessive stretch under physiological loading. Other possible mechanisms like the caveolar system [75], some flask-shaped invaginations with diameters ranging from 6 nm to 9 nm located in the cell membrane, and the fluid loss induced by large cell deformation [76] may also play important roles in reducing membrane strains during cartilage compression.

## 4. Molecule Initiation of the Formation of Membrane Ruffles

### 4.1. The Growth Factor-Dependent Mode

#### 4.1.1. Epidermal Growth Factor (EGF)

**Figure 3:**
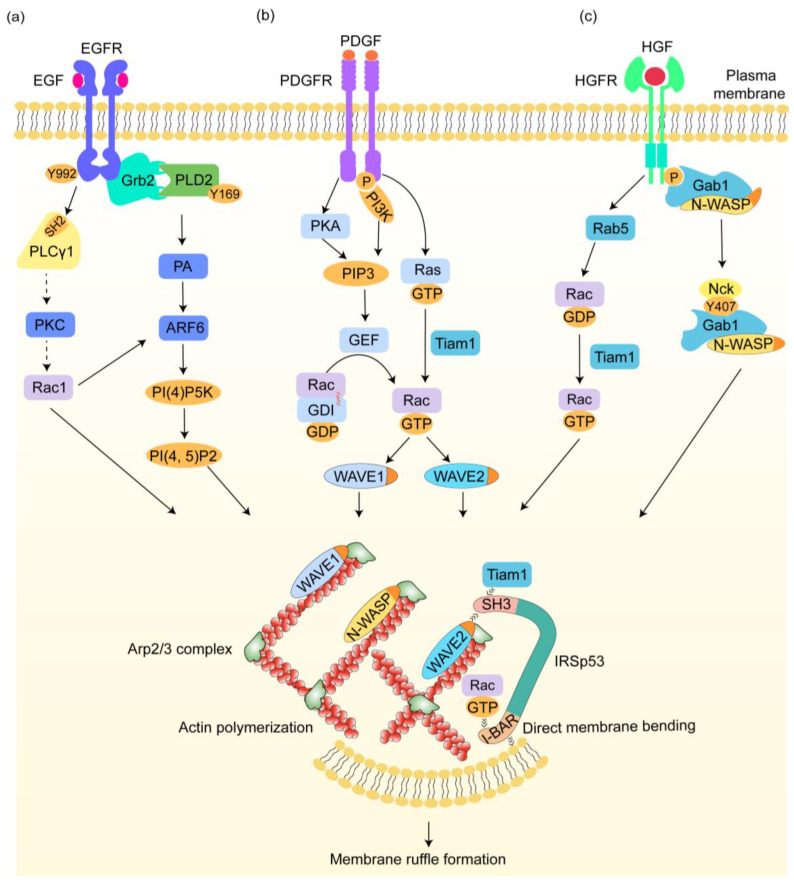
The initiation mechanism of membrane ruffles induced by growth factors. (**a**) Upon epidermal growth factor(EGF) stimulation, the receptor autophosphorylates at tyrosine 992, which facilitates phospholipase Cγ1 (PLCγ1) recruitment via its SH2 domain. PLCγ1 activation subsequently activates Rac1, which may be mediated by protein kinase C (PKC). Rac1 induces IRSp53 to bind to the Wiskott-Aldrich syndrome protein (WASP) family verprolin-homologous protein-2 (WAVE2), which activates the Arp2/3 complex, then stimulates actin polymerization and membrane ruffle formation. In addition, epidermal growth factor receptors (EGFR) activation leads to Grb2 binding, which associates with phospholipase D2 (PLD2) at the Y169 site, enhancing ruffle formation through increased phosphatidic acid (PA) production. The presence of PA also activates ADP-ribosylation factor (ARF) 6, an activator of phosphatidylinositol 4-phosphate 5-kinase (PI(4)P5K) that produces phosphatidylinositol 4,5-bisphosphate (PI(4,5)P2) and activates the Arp2/3 complex. Rac1 can also activate ARF6. (**b**) After platelet-derived growth factor (PDGF) stimulation, protein kinase A (PKA) activation facilitates phosphatidylinositol 3,4,5-trisphosphate (PIP3) accumulation. On the other hand, platelet-derived growth factor receptor (PDGFR) activation triggers phosphoinositide 3-kinase (PI3K), promoting PIP3 synthesis. PIP3 then recruits guanine nucleotide exchange factors (GEFs) to the membrane, leading to Rac release from the RhoGDI and subsequent activation. Activated Rac then binds to WAVE1. PDGF stimulation also activates Ras-GTP, which binds to Tiam1 and signals to Rac-GTP. Tiam1 interacts with IRSp53, enhancing the binding of Rac-IRSp53-WAVE2. WAVE1/2 activates the Arp2/3 complex. (**c**) Hepatocyte growth factor (HGF) activation recruits a Gab1-N-WASP complex to the membrane, with Gab1 phosphorylation at Y407 creating a docking site for Nck proteins. Nck then binds to it through the SH3 domain for N-WASP activation, which then recruits the Arp2/3 complex. Additionally, Rab5, as a downstream effector, activates Rac via Tiam1, which then induces circular dorsal ruffles (CDR) formation via Rac-IRSp53-WAVE2. Dashed arrows indicate mechanisms yet to be fully elucidated.

EGF is a heat-resistant single-chain low-molecular-weight polypeptide composed of 53 amino acid residues. EGF was discovered in 1956 and has been shown to have an effect in promoting the proliferation, growth, and differentiation of epidermal cells [77]. As shown in Figure 3a, upon binding with the receptor EFGR, the receptor conducts autophosphorylation at tyrosine 992, enabling the binding of phospholipase Cγ1 (PLCγ1) through its SH2 domain. PLCγ1 then acts as an upstream signal of Rac1. Activated Rac1 induces IRSp53 to bind to WAVE2, which then activates the Arp2/3 complex for actin assembly to generate membrane ruffles [53]. Although the mechanism of the signal transduction from PLCγ1 to Rac1 remains unknown, it is believed that PKC may mediate it [78]. In another way, after EGFR activation, the docking protein Grb2 is recruited to its cellar compartments together with Shc. Grb2 interacts with the enzyme phospholipase D2 (PLD2) at the Y169 site, which enhances membrane ruffling through the escalation of phosphatidic acid (PA) produced by PLD2. At the same time, Rac2 may play an important role in promoting this process by binding PA and its associated lipids, as well as in mapping Rac’s capabilities [79].

In the presence of PA, EGF binding also activates the ADP-ribosylation factor (ARF) 6, an endogenous activator of phosphatidylinositol 4-phosphate 5-kinase (PI(4)P5K) which catalyzes the production of PI(4,5)P2, and then activates the Arp2/3 complex for actin reorganization, thus inducing ruffle formation [4,80,81,82,83]. Moreover, ARF6 can also be activated in a Rac1-dependent manner via the ARF–guanine nucleotide exchange factors (GEFs). During the process of ruffle generation, mDia1, a downstream effector of EGF, acts as an actin nucleator, which can accelerate the polymerization of branched actin filaments induced by the Arp2/3 complex [84]. Additionally, another actin nucleator, Cobl has been found to be highly enriched in EGF-stimulated cell ruffles.

#### 4.1.2. Platelet-Derived Growth Factor (PDGF)

PDGF is a basic glycoprotein with a molecular weight of 28~35 kD, which is a dimer of two polypeptide chains (chain A and chain B) linked by a disulfide bond and was recognized as an important cell division agent stimulating the division and proliferation of vascular smooth muscle cells, fibroblasts, glial cells, and other cells [85]. Furthermore, it also has a regulatory effect on development and cell differentiation [86,87], and plays a prominent role in wound healing [88]. As a growth factor, it also induces circular dorsal ruffles like EGF (Figure 3b).

In the process of PDGF-induced membrane ruffle formation, protein kinase A (PKA) plays an important role through the modulation of 3-phosphatidylinositide (3-PI) dynamics. Briefly, after PDGF stimulation, PKA seems to be essential in the gathering and localizing of 3-PI products, phosphatidylinositol 3,4,5-trisphosphate (PIP3) in particular, at membrane ruffles [89]. Meanwhile, there is a PKA-independent signal pathway where the PDGF receptor activates PI3-kinase (PI3K) directly or indirectly and PI3K induces the synthesis of PIP3. With the presence of PIP3, GEFs are recruited to the cell membrane, which induces the exchange of GDP for GTP, thus leading to the dissociation of the RhoGDI–Rac complex and Rac activation. Activated Rac then binds to WAVE1, a scaffold protein that regulates the reorganization of actin filaments [90], thus activating the Arp2/3 complex [91], which acts as its downstream effector via its VCA domain [45,46]. The Arp2/3 complex can provide nucleation sites for the polymerization of actin filaments, leading to the formation of CDR [83,84,85,86,87,88,89].

In addition, PDGF stimulation also activates Ras-GTP, which then binds to Tiam1, facilitating the signaling cascade to Rac-GTP. Tiam1 then interacts with the SH3 domain of IRSp53. This interaction promotes the association of IRSp53 with active Rac and WAVE2. WAVE2 then activates the Arp2/3 complex, driving actin polymerization and leading to membrane ruffling [92]. In addition, during PDGF-induced dorsal ruffle formation, WIP may act as a downstream effector of activated RTK. Potential mechanisms need to be further investigated.

#### 4.1.3. Hepatocyte Growth Factor (HGF)

HGF is a growth factor derived from mesenchymal stem cells, which promotes the division and growth of a wide variety of cells, protects organs and tissues in a variety of forms ranging from paracrine and endocrine to autocrine, and plays a role in preventing tissue aging. In addition, HGF plays an important role in promoting hepatocyte regeneration while participating in normal tissue repair after injury, cell motility, tumor formation, invasion and metastasis, differentiation, and tumor vascularization [93,94].

HGF can stimulate circular dorsal membrane ruffling through a Gab1-N-WASP-dependent pathway (Figure 3c). Briefly, upon the binding of HGF to its receptor, the Gab1–N-WASP complex is recruited to the cell surface. Then, Gab1 is phosphorylated at the Y407 site, where Nck subsequently binds to it through the SH3 domain for N-WASP activation. Activated N-WASP subsequently recruits the Arp2/3 complex, which assembles actin filaments and thus generates membrane ruffles [95,96]. In addition, Rab5, known as a regulator of endocytosis, has been reported to activate Rac by modulating the trafficking of Rac and Tiam1 (its GEF) in signal transduction, thus regulating HGF-induced CDR formation via the Rac-IPSp53-WAVE2 pathway. In the regulation process, clathrin, which is fundamental for endocytosis, seems to be crucial in Rac activation induced by Rab5 [97].

### 4.2. The Growth Factor-Independent Mode

#### 4.2.1. Extracellular Calcium-Induced Mode

**Figure 4:**
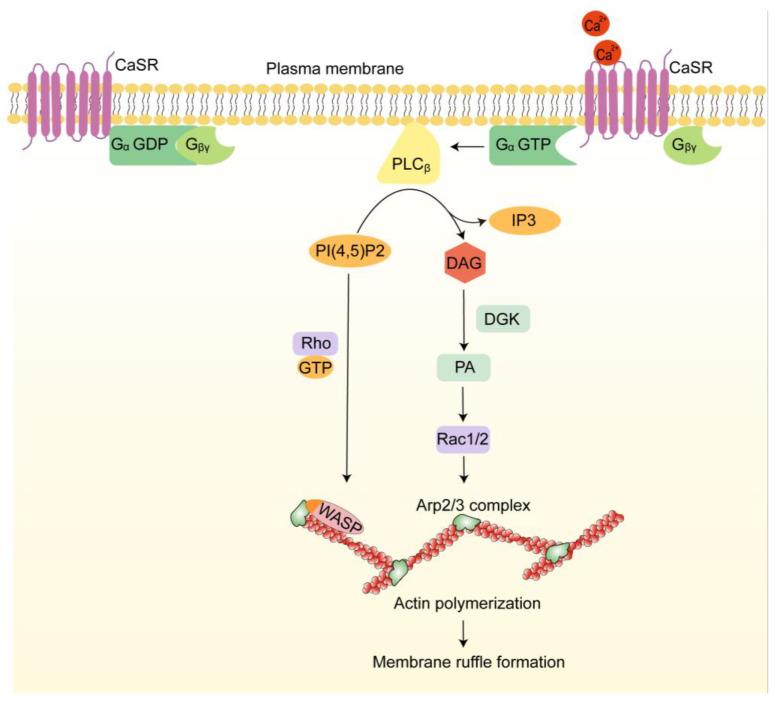
The growth factor-independent membrane ruffling triggered by extracellular calcium. In response to extracellular calcium, calcium-sensing receptor (CaSR) activates the Gα protein, leading to the dissociation of the Gβγ subunit. Active Gα-GTP then activates phospholipase C (PLC), resulting in the breakdown of PI(4,5)P2 into diacylglycerol (DAG) and inositol 1,4,5-trisphosphate (IP3). PI(4,5)P2 collaborates with active Rho GTPases to release WASP, initiating Arp2/3-mediated actin assembly. DAG is phosphorylated to form PA, which activates Rac1/2, promoting the nucleation of branched actin networks and the formation of membrane ruffles.

As mentioned above, the formation of membrane ruffles is typically initiated by the addition of growth factors in many cell types. However, professional phagocytes are unique in that they drive membrane ruffling and macropinocytosis independently of growth factors, a process triggered by extracellular calcium (Figure 4) [98,99]. Briefly, in the presence of extracellular calcium, a 7-transmembrane protein becomes activated and facilitates nucleotide exchange on its associated Gα protein. This leads to the dissociation of the Gβγ subunit from the Gα–GTP complex. The Gα subunit combined with GTP thereafter activates phospholipase C (PLC), promoting the decomposition of PI(4,5)P2 into diacylglycerol (DAG) and inositol 1,4,5-trisphosphate (IP3). PI(4,5)P2 collaborates with active Rho GTPases to release WASP, initiating Arp2/3-mediated actin assembly. DAG is phosphorylated by diacylglycerol kinase (DGK), converting into PA, which recruits GEFs and activates the small GTPase Rac1/2 [100,101]. Upon activation, Rac1/2 facilitates the nucleation of branched actin networks, thereby driving the formation of membrane ruffles.

#### 4.2.2. Phorbol Ester (PMA)

PMA, also known as tetradecanoyl phorbol acetate, is a potent activator of PKC, promoting the expression of inducible nitric oxide synthase (iNOS) in hepatocytes [102]. PMA is also a highly effective tumor promoter, demonstrated to promote the formation of skin tumors in mice [103]. Despite its high toxicity, PMA exhibits anti-leukemic and anti-neutropenia activity in humans [104]. In macrophages, treatment with PMA stimulates the phosphorylation (activation) of PKCδ, which subsequently activates slingshot homolog 1 (SSH1), leading to the dephosphorylation of cofilin at Ser-3. Then, it causes actin rearrangement and the formation of membrane ruffles [19]. Additionally, soluble N-ethylmaleimide-sensitive factor attachment protein receptor (SNARE)-mediated trafficking may be essential for delivering membrane components that enable plasma membrane remodeling during PMA-induced membrane ruffling. This is supported by the observation that inhibition of the synaptosome-associated protein 23 (SNAP23) and vesicle-associated membrane protein 4 (VAMP4) significantly reduces the formation of PMA-induced membrane ruffles [105].

#### 4.2.3. Vasodilator-Stimulated Phosphoprotein (VASP)

VASP was initially isolated from endothelial cells and platelets after stimulation with vasodilation factors such as nitric oxide donors or prostaglandins [106]; it is a substrate for cAMP- and cGMP-dependent protein kinase and has three principal phosphorylation sites, Ser157, Ser239, and Thr278 [107,108]. The localization of VASP is mainly in the focal adhesions, along stress fibers, and in the lamellipodia and filopodia [109], which bind cytoskeletal proteins and contribute to cytoskeletal dynamics in mammalian cells. For example, the phosphorylation of VASP by PKA plays a crucial role in the formation of microglial membrane ruffles and chemotaxis via the regulation of focal adhesion formation [110]. Moreover, VASP can be recruited toward the membrane by the I-BAR protein IRSp53 or lamellipodin, resulting in increased lamellipodium speed and the formation of filopodia [111]. Studies using wild-type and mutant forms of VASP in endothelial cells have shown that different subdomains of VASP play distinct roles in membrane ruffling [112]. Briefly, a proline-rich domain is necessary for its ability to bind profilin [113]. However, the binding of profilin to VASP does not appear to be essential for ruffle formation, as deletion of this proline-rich domain does not impact the formation of ruffles [112]. On the other hand, a sequence within its carboxy-terminal domain is responsible for bundle formation and VASP has the capacity to bind zyxin/vinculin-derived peptides, both of which are necessary for membrane ruffle formation. The detailed mechanisms whereby VASP causes ruffling still require further investigation.

#### 4.2.4. Lysophosphatidic Acid (LPA)

Lysophosphatidic acid (LPA) is a bioactive lipid that serves as a crucial extracellular signaling molecule, mediating various critical cellular processes, such as cell proliferation, migration, apoptosis, and morphological changes. The complex signaling pathway mediating these processes has been reviewed in detail by Geraldo et al. [114,115]. It has been found that in rat primary cultured microglia, LPA interacts with its receptor LPA_3_, activating Gα_q/11_ and PLC, which triggers the release of ATP. The released ATP then promotes membrane ruffling by activating P2Y_12_ receptors and the Gα_i/o_-PI3-K signaling pathway [115].

#### 4.2.5. Nicotinamide Adenine Dinucleotide Phosphate (NADPH) Oxidase 2 (NOX2)

Nicotinamide Adenine Dinucleotide Phosphate (NADPH) Oxidase 2 (NOX2) is a prominent member of the NADPH oxidase superfamily, known for generating superoxide (O₂^•−^), which serves as a precursor for various reactive oxygen species (ROS). Excessive production of ROS by NOX2 has been linked to multiple diseases, such as cardiovascular diseases, cancer, and neurodegenerative diseases [116,117]. Studies have demonstrated that NOX2-derived ROS are crucial for initiating membrane ruffle formation in response to PMA and M-CSF treatments. Specifically, NOX2 signaling facilitates the inactivation of phosphatase and tensin homolog (PTEN) and activates the PI3K/Akt pathway, leading to the dephosphorylation of cofilin and the subsequent formation of membrane ruffles [118].

#### 4.2.6. Thrombospondin-1 (TSP1)

Thrombospondin-1 (TSP1) is a large, homotrimeric matricellular glycoprotein that belongs to the matricellular thrombospondin protein family [119]. Each monomer of TSP1 includes an N- and C-terminal spherical domain as well as a central stalk domain, which facilitates its association with a variety of proteins. [120]. TSP1 regulates various biological processes, including cell migration [119], cell adhesion [121], inhibition of cell proliferation [122], and inhibition of tumorigenesis [123]. In macrophages, the TSP1-CD47-Nox1-cofilin signaling pathway is crucial for membrane ruffle formation and fluid-phase macropinocytosis of native LDL (nLDL). TSP1 interacts with its high-affinity receptor, CD47, to stimulate NADPH oxidase 1 (Nox1) activity. This activation leads to the dephosphorylation of cofilin at Ser-3, causing actin remodeling, membrane ruffling, and macropinocytosis. The roles of PKC and SSH1 as potential intermediaries in this pathway need further exploration [65].

In addition to the factors discussed above, others such as M-CSF [124], prolactin (PRL) [125], growth arrest-specific gene 6 (Gas6) [126], and insulin [48] may also contribute to the initiation of membrane ruffling, although research on these factors is limited. Insulin, for example, is known to induce membrane ruffling, which plays a crucial role in glucose uptake by recruiting GLUT-4 to the cell surface. Although the exact mechanism is not fully understood, the t-SNAREs syntaxin4 and SNAP-23, as well as the vesicular protein VAMP2, were observed to colocalize with GLUT4 in the ruffling regions. Similarly, PRL stimulation results in peripheral membrane ruffling, cytoskeletal reorganization, and colocalization of the Na^+^/H^+^ exchanger 1 (NHE1) with prolactin receptors (PRLRs) at the ruffles [125]. Gas6 interacts with its receptor AXL and has been identified to promote actin assembly, triggering ruffle formation [126].

#### 4.2.7. Molecules Involved in CDRs’ Ring Size Regulation

Following the initiation of CDR formation, ARAP-1, a member of the AZAP family of Arf GAPs (GTPase-activating proteins), may interact with its substrate ADP-ribosylation factor (Arf) 1 and Arf5 to regulate ring size. Briefly, ARAP-1 is dynamically localized inside the ring structure of CDRs via its multiple domains including three PH domains, one Rho GAP domain, and one Ras association (RA) domain. The Arf GAP domain of ARAP-1 can be activated by PIP3 binding and subsequently switch the GTP binding to Arf1 and Arf5 to GDP, making CDRs contract to avoid being disassembled; thus, the ring size (including the diameter of the two axes and parameters) increases [127]. Therefore, the normal size of CDRs is regulated in an ARAP1-Arf1/Arf5-dependent manner, and the depletion or overexpression of ARAP1 will lead to smaller or larger CDRs.

In summary, the formation of membrane ruffling involves a complex interplay of various regulatory proteins such as the Src family kinases, Gab1, Crk protein, SH3 domain-containing YSC84-like protein, and integrins, which play a distinct role in orchestrating cellular responses and have been already reviewed by Hoon et al. [4]. However, our understanding of the precise make-up of membrane ruffles is still rather limited. Fundamental questions regarding identifying these differentiating factors and their roles remain to be addressed in future research.

## 5. Inhibitors in the Formation of Membrane Ruffles

### 5.1. Inhibitors Related to Actin Polymerization

Cytochalasin D, an actin polymerization inhibitor, can completely block CDR formation in response to EGF and insulin, and significantly reduce growth factor-induced phosphorylated AKT levels (pAKT) in Hep3B cells [128]. Latrunculins, which bind stoichiometrically to actin monomers and prevent actin polymerization, also inhibit membrane ruffling and macropinocytosis [129]. A jasplakinolide/blebbistatin (J/B) combination inhibited actin filament turnover and myosin II, respectively, effectively blocking PDGF-induced ruffles and macropinocytosis in macrophages. Additionally, microtubule dynamics have been shown to affect actin dynamics during ruffle formation. Nocodazole, a microtubule-depolymerizing agent, inhibits EGF-mediated CDR formation and disrupts Akt phosphorylation [130]. Furthermore, microtubule-stabilizing agents like Taxol inhibit the formation of linear membrane ruffles.

Moreover, the reorganization of the actin cytoskeleton to generate membrane ruffles and actin foci depends on the activity of multiple ABPs, such as the WASP–Arp2/3 complex [131], cortactin [131,132], paxillin [133,134], and vinculin [126]. It has been shown that depleting these proteins inhibits the formation of CDR [128], though the mechanisms remain incompletely defined.

### 5.2. Inhibitors of Sodium/Hydrogen Exchangers (NHEs)

Inhibitors of NHEs, such as amiloride and ethylisopropyl amiloride (EIPA), prevent the induction of membrane ruffling [135]. Amiloride was shown to inhibit macropinocytosis by decreasing submembranous pH and blocking the Rac1 and Cdc42 pathway [136].

### 5.3. Inhibitors of Protein Kinase

Wortmannin, which specifically inhibits PI3-kinase at low concentrations, has been shown to inhibit both peripheral and dorsal ruffles [25]. Similarly, LY294002 also acts as a PI3-kinase inhibitor and blocks CDR formation [137]. TGX221, a p110β-specific inhibitor, completely blocked CDR formation and significantly attenuated growth factor-induced pAKT in Hep3B cells [128]. The type Iα phosphatidylinositol phosphate kinase (PIPKIα) isoform is localized to PDGF-induced membrane ruffles, and expression of a dead PIPKIα kinase blocks the formation of membrane ruffles. IPA-3, an inhibitor that targets p21-activated kinase 1 (PAK1), is employed to inhibit membrane ruffling [135].

### 5.4. Others

Depletion of membrane cholesterol using methyl-β-cyclodextrin (mβCD) inhibits PMA-induced membrane ruffle formation in A431 cells [138]. The underlying mechanism involves cholesterol depletion, leading to the redistribution of phosphoinositides in the membrane, which affects the localization of Rac1, ARF6, and other factors [139]. In addition, it has been demonstrated that imipramine blocks the formation of PMA-induced membrane ruffles by inhibiting the molecules involved in ruffle formation, except for the Ras/MEK/ERK, PKCδ, and PI3K pathways. In contrast, phenoxybenzamine, a non-selective and irreversible alpha blocker, disrupts multiple signaling pathways involved in both the early stages of ruffle formation and the plasma membrane processes that occur downstream of cup formation [129]. Furthermore, Snx33, a curvature-sensitive BAR domain protein, has been found to inhibit actin polymerization and ruffling [58].

## 6. Visualization and Quantification Technologies for Membrane Ruffles

### 6.1. Phase-Contrast Microscopy

**Figure 5:**
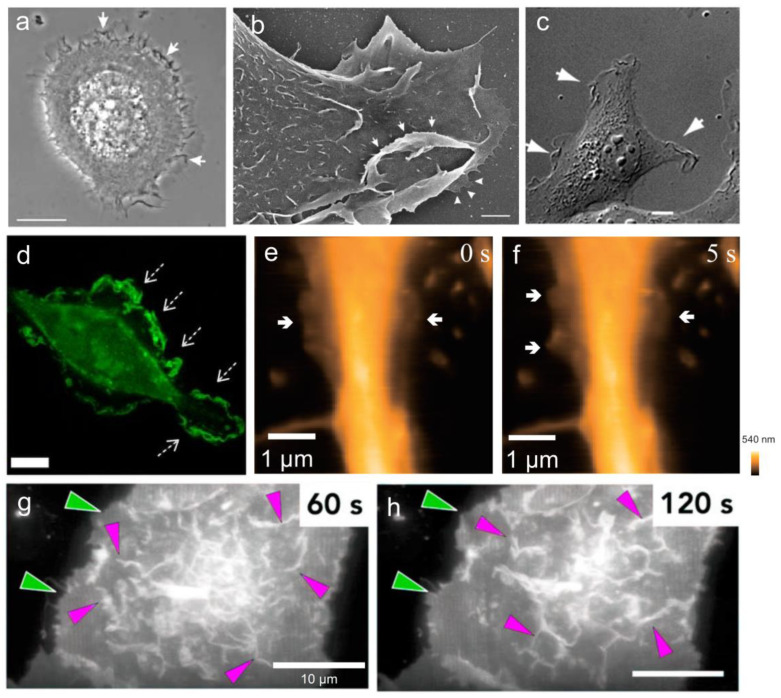
The micrographs of membrane ruffles visualized by various techniques. Membrane ruffles (white arrows) formed in keratinocytes captured by phase-contrast microscopy (**a**) and scanning electron microscopy (SEM) (**b**) [44]. Cos1 cells stimulated with epidermal growth factor (EGF) after 5 min imaged by differential interference contrast (DIC) microscopy (**c**) [144] Human monocyte-derived macrophage imaged by confocal microscopy (**d**) [145]. Arrows indicate the membrane ruffle area. Time-lapse images of live hippocampal neurons visualized by high-speed atomic force microscopy (HS-AFM) (**e**,**f**) [146]. The arrows points to the thin, sheet-like ruffling. Dorsal membrane ruffles (magenta arrows) formed in human retinal pigmented epithelial (RPE1) cells imaged by lattice light-sheet microscopy (LLSM) (**g**,**h**) [83]. The green arrows indicate the increased protrusion. Scale bars: 10 μm in (**a**,**c**,**d**,**g**,**h**); 1 μm in (**b**,**e**,**f**).

Phase-contrast microscopy is a widely used technique for observing cellular ruffles with high contrast in living cells. In 1970, Abercrombie and his colleagues [140,141] captured time-lapse films of fibroblast ruffles using a phase-contrast objective and found the ruffles formed near the lamella front edge, which appears as a dark winding line in the plan view. Borm et al. [44] also employed phase-contrast microscopy to visualize the motile keratinocytes (Figure 5a), revealing membrane ruffles as distinct dark waves at the leading edge moving centripetally toward the main cell body, where they eventually fade away. In addition, Bernitt et al. [142] utilized phase-contrast microscopy to capture sequential time-lapse images of membrane ruffles during their spontaneous formation processes, which initiate from localized points on the dorsal membrane, expand, contract, and collapse to a single focal point, finally forming macropinosomes. However, phase-contrast microscopy has limitations in imaging resolution [143].

### 6.2. Differential Interference Contrast (DIC) Microscopy

Differential interference contrast (DIC) microscopy enables high-contrast, low-phototoxicity, and three-dimensional imaging, making it well-suited for capturing membrane ruffles in live cells (Figure 5c) [144]. Patel et al. [3] utilized a Zeiss Axiovert 200M microscope equipped with DIC and epifluorescence optics to capture DIC time-lapse movies, observing prominent membrane ruffles in macrophages stimulated with PMA and LPS. In a study performed by Schlam et al. [145], DIC microscopy was employed to observe the actin dynamic response of macrophages treated with or without gliotoxin. The time-lapse imaging revealed that, in response to the toxin, macrophages ceased to ruffle, underwent abrupt membrane retraction, and exhibited reduced effectiveness in phagocytosing large targets. However, the imaging resolution of DIC microscopy is still limited by numerical aperture (NA) and wavelength [147].

### 6.3. Scanning Electron Microscopy (SEM)

SEM allows for the elucidation of the ultrastructure of membrane ruffles. In the early 1970s, the electron micrograph of membrane ruffles in fibroblasts was unveiled [148]. After that, SEM micrograph of ruffles at the leading edge or dorsal surface were observed in many other cell types, such as chick heart fibroblast [1], bone marrow-derived macrophages (BMDMs) [149,150], human epidermal keratinocytes (Figure 5b) [44], RAW264.7 mouse macrophages [3], and MCF-7 human breast adenocarcinoma cells [151]. Recently, Ahn et al. [152] published a comprehensive SEM protocol to visualize and quantify membrane ruffle formation in RAW 264.7 macrophages, in which they captured images of different morphological stages of micropinocytosis including large sheet-like ruffles, circular C-shaped ruffles, and macropinocytotic cups. Undoubtedly, SEM provides perfect resolution for surface imaging. However, the thorough dehydration of samples and the vacuum environment required for imaging restricts the examination of live cells [153].

### 6.4. Confocal Laser Scanning Microscopy (CLSM)

Confocal laser scanning microscopy (CLSM) is particularly well suited for fluorescence imaging of membrane ruffles [128,154], especially live cell imaging, as it allows for the visualization of three-dimensional and dynamic changes in ruffles. Pittman et al. [2] transfected MDA-MB-231 cells with eGFP-F-tractin to label actin-rich peripheral ruffles, obtained z-stack images at 1 min intervals by using Leica TCS SP8, then calculated the ruffle index. In addition, Schlam et al. performed real-time imaging of macrophage electro-transfected with Akt(PH)-GFP to label PtdIns(3,4,5)P3. They found that PtdIns(3,4,5)P3 was highly accumulated in the region of membrane ruffles generated by macrophages (Figure 5d) [145]. However, the prolonged exposure of cells to lasers may lead to photobleaching or induce phototoxic effects, potentially impacting the cells’ viability and behavior [155].

Quantitative analysis of the ruffle area is usually performed in Fiji/ImageJ software (https://imagej.net/software/fiji/, accessed on 9 October 2024) [154]. Condon et al. [156] developed a ruffle quantification ImageJ macro for automatically quantifying dorsal ruffles from 3D microscope images, enabling batch measurements of ruffle area and intensity.

### 6.5. High-Speed Atomic Force Microscopy (HS-AFM)

Atomic force microscopy (AFM) is a powerful tool for studying cytoskeletal dynamics in living cells under nearly physiological conditions [157,158]. In recent years, the emergence of high-speed-AFM (HS-AFM) has significantly improved scanning speed, reaching two orders of magnitude faster than that of conventional AFM. The development of HS-AFM has made it possible to elucidate the microstructure and dynamic changes in the cell membrane. Shibata et al. [146,159] utilized an ultra-thin (~5 nm) and elongated (~3 µm) amorphous carbon tip affixed to a cantilever, enabling high-resolution, nanometer-scale visualization of dynamic cellular processes. This technique allowed for real-time observation of filopodia, membrane ruffles (Figure 5e,f), pit formation, and endocytosis in live COS-7 and HeLa cells, as well as hippocampal neurons, with second-scale temporal precision. However, the scanning range of HS-AFM remains to be improved and the impact of a tip sample should be taken into account [160].

### 6.6. Lattice Light-Sheet Microscopy (LLSM)

Recently, LLSM has emerged as a highly effective tool for exploring complex three-dimensional morphologies, providing both high spatial and temporal resolution [161,162]. More recently, Quinn et al. [163] utilized this technology to record the complete evolution of membrane ruffles and the mechanism by which these structures form into macropinosomes. Condon et al. [7] employed LLSM for very rapid, high-resolution imaging of LPS-stimulated live macrophages and revealed the formation of tent pole ruffles, the crossover and twisting of which form the macropinosome. In another study, Leyden et al. [83,164] combined LLSM with optogenetic activation to visualize dynamic and transient Rac1 ruffles on the dorsal surface of human retinal pigmented epithelial (RPE1) cells (Figure 5g,h). Notably, LLSM minimizes phototoxic effects on living cells in comparison to traditional optical technologies, allowing for extended imaging sessions without significant sample damage [165,166]. However, the high magnification, combined with a limited field of view, make it difficult to efficiently identify and select specific regions [167].

All of the visualization techniques of membrane ruffles are summarized in Table 1.

## 7. Conclusions

Although a number of experimental results have been reported on the structure, function, formation, and visualization of membrane ruffles, our understanding of the ruffles characteristics is still limited. A recent study performed by Pittman et al. [2] has extended the function of membrane ruffling from cell motility, micropinocytosis, and receptor internalization to extracellular fluid viscosity sensing in a 2D culture conditions. Whether its viscosity sensing ability works in a 3D microenvironment such as in atherosclerotic lipid-rich plaques or in tumors and the involved underlying signal cascades have yet to be explored. The polarization of NHE1, the activation of the transient receptor potential cation vanilloid 4 (TRPV4), the subsequent calcium influx, and the downstream RhoA-based contractility [168] may be potential signal candidates for further investigation.

Multiple proteins and factors have been reported to participate in the initiation and formation of membrane ruffles, which is a sophisticatedly regulated event. However, the key determinants for the structure differences in ruffles are still unknown. For professional phagocytes, membrane ruffling does not even rely on growth factor stimulation. Dendritic cells (DCs) and macrophages, in particular, perform macropinocytosis continuously without the need for external stimuli. This process is highly active, with macrophages internalizing their entire cell surface every 33 min, while human DCs take up >1000 μm of extracellular fluid—roughly 40% of the volume of the cell—every hour [98]. The mechanisms of ruffle formation in constitutive-induced macropinocytosis are not yet fully understood. However, it should be noted that the concentration of calcium in the extracellular environment must be approximately four orders of magnitude higher than that of intracellular calcium to trigger the downstream signaling pathways to initiate membrane ruffle formation [99]. Such high extracellular calcium concentrations indeed exist in some pathological conditions. For example, in the microenvironment of the atherosclerotic plaque, the concentration of calcium is much higher than the normal tissue due to the deposits of calcium salt. In this case, could the plaque calcium behave as a chemotactic agent that initiates ruffle formation and orchestrates the migration of monocytes and macrophages to the subendothelial space? This should be further investigated. As it is a downstream event, calcium-induced frequent membrane ruffling may affect the effective adhesion of macrophages and foam cells to the substrate, which may explain to some extent the lower migrative ability of these cells and their retention in the plaque.

## Figures and Tables

**Table 1:** The visualization techniques of the membrane ruffles.

Visualization Technique	For Live Cell Imaging?	Advantages	Limitations	References
Phase-contrast microscopy	Yes	High-contrast, no requirement of stains or dyes, and no effect on cell viability	Imaging resolution limited, and suffers from the halo and shade-off artifacts	[44,140,141,142,143]
Differential interference contrast (DIC) microscopy	Yes	Higher-contrast, low-phototoxicity, and label-free imaging, and fine spatial resolution	Imaging resolution limited by numerical aperture and wavelength	[3,144,145,147]
Scanning electron microscopy (SEM)	No	Subnanometer resolution, providing ultrastructure of cell morphology	Not applicable to live cells, and complex sample preparation process	[1,3,44,148,149,150,151,152,153]
Confocal laser scanning microscope (CLSM)	Yes	Fluorescence imaging, fixed cell imaging, live cell imaging, simple and easy to implement, and captures three-dimensional images	Photobleaching or phototoxic effects, impacts cells’ viability and behavior, and comparatively lower imaging resolution along the *z*-axis	[2,128,145,154,155,156]
High-speed atomic force microscopy (HS-AFM)	Yes	Provides nanometer-resolution and subsecond frame rate, and imaging under nearly physiological conditions	The scanning range remains to be improved, and the impact of the tip sample should be taken into account	[146,157,158,159,160]
Lattice light-sheet microscopy (LLSM)	Yes	Studies 3D dynamics in biological samples at subcellular scales, real-time and multi-dimensional imaging of live cells, and very low phototoxicity and photobleaching	Challenges in quickly selecting specific cells or regions of interest across a large sample	[7,83,161,162,163,164,165,166,167]

## Data Availability

Not applicable.
